# Respiratory Syncytial Virus and Influenza Infections in Children in Ulaanbaatar, Mongolia, 2015–2021

**DOI:** 10.1111/irv.13303

**Published:** 2024-05-16

**Authors:** Lien Anh Ha Do, Naranzul Tsedenbal, Chimidregzen Khishigmunkh, Bazarkhuu Tserendulam, Lkhagvadorj Altanbumba, Dashtseren Luvsantseren, Munkhchuluun Ulziibayar, Bujinlkham Suuri, Dorj Narangerel, Bilegtsaikhan Tsolmon, Sodbayar Demberelsuren, Casey L Pell, Sam Manna, Catherine Satzke, Cattram Nguyen, Tuya Mungun, Claire von Mollendorf, Darmaa Badarch, Kim Mulholland

**Affiliations:** ^1^ New Vaccines Group Murdoch Children's Research Institute Melbourne Australia; ^2^ Department of Paediatrics The University of Melbourne Parkville Australia; ^3^ Virology Department National Center of Communicable Diseases Ulaanbaatar Mongolia; ^4^ National Center for Communicable Diseases Ministry of Health Ulaanbaatar Mongolia; ^5^ Medical Department Mongolian National University of Medical Sciences Ulaanbaatar Mongolia; ^6^ Expanded Program on Immunization World Health Organization Ulaanbaatar Mongolia; ^7^ Translational Microbiology Group Murdoch Children's Research Institute Melbourne Australia; ^8^ Department of Microbiology and Immunology at the Peter Doherty Institute for Infection and Immunity The University of Melbourne Melbourne Victoria Australia; ^9^ Infectious Disease Epidemiology & International Health London School of Hygiene and Tropical Medicine London UK

**Keywords:** influenza virus, lower respiratory tract infections, respiratory syncytial virus

## Abstract

**Background:**

Data available for RSV and influenza infections among children < 2 years in Mongolia are limited. We present data from four districts of Ulaanbaatar from April 2015 to June 2021.

**Methods:**

This study was nested in an enhanced surveillance project evaluating pneumococcal conjugate vaccine (PCV13) impact on the incidence of hospitalized lower respiratory tract infections (LRTIs). Our study was restricted to children aged < 2 years with arterial O_2_ saturation < 93% and children with radiological pneumonia. Nasopharyngeal (NP) swabs collected at admission were tested for RSV and influenza using qRT‐PCR. NP swabs of all patients with radiological pneumonia and of a subset of randomly selected NP swabs were tested for *S. pneumoniae* (*S.p*.) by qPCR and for serotypes by culture and DNA microarray.

**Results:**

Among 5705 patients, 2113 (37.0%) and 386 (6.8%) had RSV and influenza infections, respectively. Children aged 2–6 months had a higher percentage of very severe RSV infection compared to those older than 6 months (42.2% versus 31.4%, *p*‐value Fisher's exact = 0.001). *S.p*. carriage was detected in 1073/2281 (47.0%) patients. Among *S.p*. carriage cases, 363/1073 (33.8%) had *S.p*. and RSV codetection, and 82/1073 (7.6%) had *S.p*. and influenza codetection. *S.p*. codetection with RSV/influenza was not associated with more severe LRTIs, compared to only RSV/influenza cases.

**Conclusion:**

In Mongolia, RSV is an important pathogen causing more severe LRTI in children under 6 months of age. Codetection of RSV or influenza virus and *S.p*. was not associated with increased severity.

## 1 Introduction

Globally respiratory syncytial virus (RSV) is the leading cause of lower respiratory tract infections (LRTIs) in young children under 2 years of age, with influenza also recognized as an important viral cause of childhood pneumonia [1, 2]. Additionally, *Streptococcus pneumoniae* (*S.p*.) remains an important bacterial pathogen causing pneumonia in children, particularly in low‐middle income countries (LMICs), despite pneumococcal conjugate vaccines (PCV) being available since 2000 [3]. Nasopharyngeal carriage of *S.p*. is recognized as a prerequisite for pneumococcal pneumonia [4]. PCV introduction has been shown to reduce pneumococcal serotypes targeted by a specific PCV but also accompanied by a replacement of nonvaccine serotype carriage [5]. Synergistic effects between RSV or influenza virus and *S.p*. carriage have been previously reported in clinical settings and experimental models [6, 7, 8, 9, 10, 11]. However, these effects varied widely across different clinical settings [6, 7, 8, 9, 10, 11].

In a cold, LMIC setting with a high risk of air pollution such as Mongolia, there are limited data on epidemiological and clinical characteristics of RSV and influenza infections as well as the role of codetections of those viruses and *S.p*. carriage in young children. These data provide a valuable information on the synergistic relationship between *S.p*. and RSV or influenza virus.

The largest measles outbreak in Mongolia during the last 20 years occurred in 2015–2016 [12, 13]. The measles outbreak had a biphasic epidemic pattern. During the second phase of the outbreak, there was a 10‐fold rise in measles mortality which coincided with a peak in RSV disease [14]. This leads to a question on how the measles infection contributed to the epidemiological curve of RSV/influenza infection during that time.

In this study, we present the first and comprehensive 6‐year data on clinical and epidemiological characteristics of RSV/influenza infection and their relationship with pneumococcus and measles virus in hospitalized children under 2 years of age with LRTIs in four districts in Ulaanbaatar city, Mongolia.

## 2 Methods

### 2.1 Study Setting

A study using the WHO LRTI surveillance system was conducted to evaluate PCV13 impact on pneumonia among children 2–59 months of age, before and after phased PCV13 introduction, from April 2015 to June 2021 in four districts in Ulaanbaatar (PCV Impact Evaluation Study) [15].

Our current study (the RSV/Influenza sub‐study) is nested within the setting of the PCV Impact Evaluation Study, with a focus on children under 2 years of age.

### 2.2 Study Population

All children enrolled in the PCV Impact Evaluation study who were aged 2–23 months and met the below case definitions were eligible for inclusion in the RSV/Influenza substudy:

- Severe LRTI: Acute illness associated with cough or breathing difficulty AND fast breathing (respiratory rate > 50) AND arterial oxygen saturation (SaO_2_) less than 93% [16].
- Very severe LRTI: Acute illness associated with cough or breathing difficulty, AND fast breathing (respiratory rate > 50) AND SaO_2_ less than 90% or altered consciousness [16].
- Radiological pneumonia: Defined by WHO criteria [17].

### 2.3 Data and Samples' Collection

Clinical, epidemiological, demographic data and samples collected as part of the PCV Impact Evaluation Study were used to describe the epidemiological characteristics of LRTIs associated with RSV and influenza in children under 2 years of age.

We examined the relationship between RSV/influenza and *S.p*., by investigating the association between the presence of RSV/influenza and *S.p*. in the nasopharynx, the density of the pathogens in question and the relationship with severity.

We retrospectively reviewed medical archives to confirm measles infection status among our study participants during the 2015/2016 outbreak, to examine the relationship between measles infections and RSV/influenza severity. These reported measles cases were either clinically diagnosed or laboratory confirmed.

Details of data and sample collection for the PCV Impact Evaluation Study were previously described [15]. Samples were collected and tested for *S.p*. for a subgroup of LRTI cases comprising all radiologically confirmed pneumonia cases plus an additional number of randomly selected LRTI cases. We used *lytA* qPCR, culture and microarray to detect and serotype *S.p*. [5].

RSV and influenza virus PCR testing was performed for all nasopharyngeal (NP) swabs selected for the RSV/Influenza substudy, using previously published protocols [18].

### 2.4 Data Analysis

#### 2.4.1 Clinical and Demographic Characteristics of RSV/Influenza Infections

Continuous variables were summarized using medians and interquartile ranges (IQRs). Categorical variables were summarized with frequency counts and percentages. The Mann–Whitney *U* test was used to compare continuous variables, and the Fisher's exact test was used for comparisons of categorical variables between groups.

#### 2.4.2 Epidemiological Risk Factors Associated With Very Severe RSV/Influenza LRTIs

Univariable and multivariable logistic regression models were used to explore potential epidemiological risk factors for very severe LRTI associated with RSV or influenza. The following potential risk factors were examined in the two models: age, prevalence of pneumococcal carriage, current breastfeeding, indoor smoke exposure (e.g., fuel type used at home), cigarette smoking indoors, number of children < 5 years in household and whether children were sent to a child‐care center. These risk factors were based on previous RSV epidemiological studies [19]. Separate analyses were performed for RSV and influenza infections.

#### 2.4.3 Relationship Between Measles Infections and RSV/Influenza Severity

Pearson's correlation coefficient was used to assess the strength of linear relationship between the monthly case number of measles and of RSV/influenza infections.

#### 2.4.4 Association Between *S.p.* and RSV/Influenza Infections

For cases with data on both *S.p*. and viruses, we examined: (i) the association between *S.p*. carriage and RSV/influenza detection using Fisher's exact test; (ii) the correlation between density of *S.p*. carriage and RSV/influenza viral load using Pearson correlation coefficients (viral load and *S.p*. density data were log‐transformed prior to analysis); (iii) the relationship between disease severity and codetection of *S.p*. with RSV or influenza using Fisher's exact tests. The outcome variable was disease severity (severe LRTI or very severe LRTI) according to case definitions above. The exposure variable was codetection (codetection = 1 if detected with *S.p*. and either RSV or influenza; codetection = 0 otherwise).

#### 2.4.5 Relationship Between Disease Severity and Viral Load or *S.p.* Density

This analysis only included those who were positive for RSV/influenza virus and *S.p*. Disease severity was defined as above (i.e., severe LRTI or very severe LRTI). Viral load and *S.p*. density data were log‐transformed prior to analysis. This analysis was done separately for RSV and influenza. Mann–Whitney *U* test was used to examine the differences in viral load or *S.p*. density between disease severity outcomes. Viral load and *S.p*. density data were presented as medians and interquartile ranges.

All statistical analyses were implemented using Stata version 16.0 (StataCorp LP, College Station, TX, USA). All statistical tests were conducted at the two‐tailed 5% significance level.

## 3 Results

### 3.1 Clinical and Demographic Characteristics of All LRTIS and LRTIs Associated With RSV and Influenza Infections

During the period April 2015–June 2021, there were 8325 children enrolled in the PCV Impact Evaluation Study who met the case definition criteria for this study. Over two‐thirds (5705/8325, 69%) of these children had NP swabs collected. Swab collection was not possible in some cases due to limited study staff numbers at the beginning of the surveillance, delays in enrolment or where patients refused nasopharyngeal swab collection.

Among the 5705 children, 3198 (56.1%) were male, and 1528 (26.7%) were 2–6 months of age. The median age was 10 months (IQR 6–16 months), and the median duration of hospitalization was 6 days (IQR 5–8 days).

RSV and influenza detection rates were 37.0% (2113/5705) and 6.8% (386/5705), respectively. Of RSV positive cases, 55.3% (1169/2113) was RSV A, 42.9% (907/2113) was RSV B, and 1.8% (37/2113) had both RSV A and B. For influenza infection, 65.2% (252/386) of cases were due to influenza A, 34.2% (132/386) to influenza B and 0.5% (2/386) to both influenza A and influenza B. There were 1.2% (71/5705) cases coinfected with both RSV and influenza (Table 1).

**TABLE 1:** Clinical and demographic characteristics of study participants by districts during the study period (April 2015–June 2021).

Category	Subcategories *n* (%)	CHD *N* = 1220	BZ *N* = 2028	SK *N* = 1506	SB *N* = 951	All districts *N* = 5705
Sex	Male	667 (54.7)	1195 (58.9)	813 (54.0)	523 (55.0)	3198 (56.1)
Age group	< 3 m	35 (2.9)	70 (3.5)	63 (4.2)	24 (2.5)	192 (3.4)
	3–6 m	285 (23.4)	503 (24.8)	353 (23.4)	195 (20.5)	1336 (23.4)
	7–11 m	370 (30.3)	622 (30.7)	489 (32.5)	278 (29.2)	1759 (30.8)
	12–24 m	530 (43.4)	833 (41.1)	601 (39.9)	454 (47.7)	2418 (42.4)
Number of siblings	No siblings	788/1199 (65.7)	1333/2005 (66.5)	932/1485 (62.8)	597/938 (63.7)	3650/5627 (64.9)
	Any siblings	411/1199 (34.3)	672/2005 (33.5)	553/1485 (37.2)	341/938 (36.4)	1977/5627 (35.1)
Crowding (People/room)^a^	≤ 3	241/1194 (20.2)	615/1992 (30.9)	360/1469 (24.5)	297/934 (31.8)	1513/5589 (27.1)
	> 3	953/1194 (79.8)	1377/1992 (69.1)	1109/1469 (75.5)	637/934 (68.2)	4076/5589 (72.9)
Smoker in the home^a^	Yes	105/1198 (8.8)	217/1997 (10.9)	182/1485 (12.3)	97/937 (10.4)	601/5617 (10.7)
Fuel for cooking^a^	Electricity/gas	212/1190 (17.8)	940/1993 (47.2)	379/1481 (25.6)	416/934 (44.5)	1947/5598 (34.8)
	Wood/coal	978/1190 (82.2)	1053/1993 (52.8)	1102/1481 (74.4)	518/934 (55.5)	3651 (65.2)
Khoro type^a^	Apartment	196/1200 (16.3)	879/2004 (43.9)	346/1485 (23.3)	393/938 (41.9)	1814/5627 (32.2)
	Ger	498/1200 (41.5)	692/2004 (34.5)	774/1485 (52.1)	268/938 (28.6)	2232/5627 (39.7)
	Mixed	506/1200 (42.2)	433/2004 (21.6)	365/1485 (24.6)	277/938 (29.5)	1581/5627 (28.1)
Household income^a^	Above minimum	708/1058 (66.9)	1156/1927 (60.0)	690/1437 (48.0)	485/889 (54.6)	3039/5311 (57.2)
	At/below minimum	350/1058 (33.1)	771/1927 (40.0)	747/1437 (52.0)	404/889 (45.4)	2272/5311 (42.8)
Household member treated for tuberculosis^a^		17/1155 (1.5)	36/1984 (1.8)	14/1467 (1.0)	7/930 (0.8)	74/5536 (1.3)
Asthma^a^		112/1196 (9.4)	124/1977 (6.3)	125/1475 (8.5)	74/933 (7.9)	435/5581 (7.8)
Malnutrition^a^		141/1220 (11.6)	134/2027 (6.6)	141/1506 (9.4)	60/951 (6.3)	476/5704 (8.3)
History of measles infections^a^		15 (1.2)	53 (2.6)	35 (2.3)	13 (1.4)	116/5705 (2.03)
Length of hospital stay (days)^a^	> 7	677 (55.5)	1139 (56.2)	568 (37.7)	413 (43.4)	2797 (49.0)
	≤ 7	543 (44.5)	889 (43.8)	938 (62.3)	538 (56.6)	2908 (51.0)
Previous admission for pneumonia^a^		500/1198 (41.7)	614/2000 (30.7)	479/1479 (32.4)	242/927 (26.1)	1835/5604 (32.7)
Antibiotic given in hospital^a^		1195/1210 (98.8)	1809/1965 (92.1)	1420/1496 (94.9)	780/936 (83.3)	5204/5607 (92.8)
O_2_ supplementation^a^		662/1219 (54.3)	732/2021 (36.2)	678/1502 (45.1)	330/949 (34.8)	2402/5691 (42.2)
Hypoxia (O_2_ saturation < 90)^a^		463 (38.0)	680 (33.5)	515 (34.2)	303 (31.9)	1961 (34.4)
Severe LRTIs		756 (62.0)	1122 (55.3)	984 (65.3)	683 (71.8)	3545 (62.1)
Very severe LRTIs		365 (29.9)	425 (21.0)	474 (31.5)	232 (24.4)	1496 (26.2)
Xray (+)^a^		274/1151 (23.8)	242/1698 (14.3)	591/1429 (41.4)	143/797 (17.9)	1250/5075 (24.6)
Died during admission^a^		2 (0.2)	5 (0.3)	5 (0.3)	5 (0.5)	17 (0.3)
RSV positive^a^	RSV positive	482 (39.5)	763 (37.6)	532 (35.3)	336 (35.3)	2113 (37.0)
	RSV A	258 (21.2)	397 (19.6)	341 (22.6)	173 (18.2)	1169 (20.5)
	RSV B	213 (17.5)	354 (17.5)	184 (12.2)	156 (16.4)	907 (15.9)
	RSV A and B	11 (0.9)	12 (0.6)	7 (0.5)	7 (0.7)	37 (0.6)
Influenza positive^a^	Influenza positive	73 (6.0)	123 (6.1)	126 (8.4)	64 (6.7)	386 (6.8)
	Influenza A	41 (3.4)	92 (4.5)	88 (5.8)	31 (3.3)	252 (4.4)
	Influenza B	32 (2.6)	31 (1.5)	38 (2.5)	31 (3.3)	132 (2.3)
	Influenza A and B	0 (0)	0 (0)	0 (0)	2 (0.21)	2 (0.04)
RSV and influenza coinfection		18 (1.5)	22 (1.1)	15 (1.0)	16 (1.7)	71 (1.2)
*S.p*. carriage^a^		254/489 (51.9)	197/487 (40.5)	464/921 (49.6)	158/384 (41.2)	1073/2281 (47.0)
*S.p*. carriage vaccine type		114/445 (25.6)	82/447 (18.3)	199/856 (23.3)	85/361 (23.6)	480/2109 (22.8)

*Note:* SaO_2_: Oxygen saturation; Xray(+): confirmed radiological pneumonia cases; “History of measles infections” refers to cases that had measles confirmed (clinically or by laboratory) during the last year before the enrolment into the study.

Abbreviations: BZ, Bayanzurkh district; CHD, Chingeltei district; SB, Sukhbaatar district; SK, Songinokhairkhan district.

^a^ Significantly different between districts, Mann–Whitney *U p*‐value or Fisher exact test *p*‐value comparing between the continuous variables or between the dichotomous variables (*p*‐value < 0.05).

RSV patients were significantly younger than influenza patients (median age 9 months [IQR 5–16 months] versus 12 months [IQR 8–18 months], respectively, Mann–Whitney *p*‐value < 0.001). Children 2–6 months had a higher percentage of very severe RSV infection compared to those older than 6 months (34.9% [244/699] versus 24.0% [340/1414], respectively, *p*‐value = 0.001).

There were 1961/5705 (34.3%) patients with hypoxia (SaO_2_ < 90%) (Table 1). There was no significant difference in the percentage of hypoxic cases among RSV infections versus those among influenza cases (749/2105; 35.6% versus 130/383; 33.9%, Fisher exact *p*‐value = 0.6).

Social living conditions, underlying medical issues, RSV or influenza detection rates and LRTIs severity differed between the districts (Table 1).

### 3.2 RSV, Influenza Seasons and Their Subgroups

The peaks of RSV and influenza virus detection coincided with the peak of LRTI cases from the surveillance project (Figure S1). The influenza peaks usually coincided with the lowest temperatures in the winter, while RSV peaks was usually preceded the lowest temperature (Figure S2). RSV and influenza case numbers had a strong positive correlation with the reduction of environmental temperature, Pearson *r*(98) = −0.5, *p* < 0.0001 and Pearson *r*(98) = −0.5, *p* < 0.0001, respectively.

Before the onset of the COVID‐19 pandemic (i.e., April 2015–March 2020), RSV and influenza seasons started between October/November each year, peaked in December/January and ended in April. From April 2020, the number of RSV and influenza cases was reduced substantially.

**FIGURE 1:**
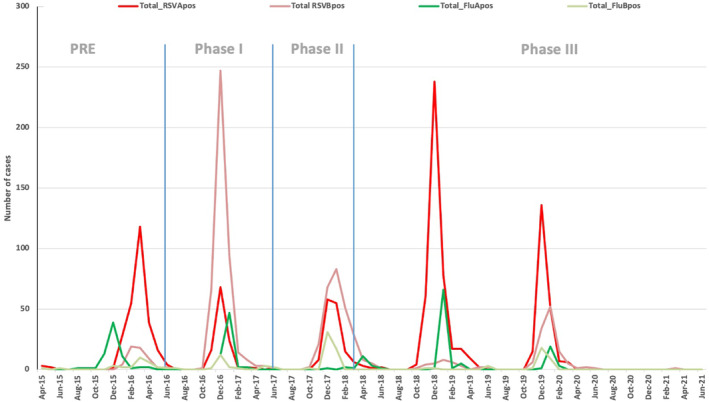
RSV and influenza subgroups during the study period. Vertical lines are the PCV introduction phased times. Pre: before PCV13 introduced. Phase I: when PCV13 introduction started in SK and SB. Phase II: when PCV13 introduction started in BZ. Phase III: when PCV13 introduction started in the rest of UB including CHD (SK: Songinokhairkhan, SB: Sukhbataar, BZ: Bayanzurkh, CHD: Chingeltei).

RSV B was dominant in two seasons (October 2016–February 2017 and October 2017–February 2018), while RSV A was dominant in three seasons. Influenza B was only dominant in the October 2017–February 2018 season, while influenza A was dominant in the other three seasons (Figure 1). Of note, there were significantly more severe LRTI cases among the RSV A cases than among the RSV B cases (816/1169, 70% versus 561/911, 62%, Fisher exact *p*‐value < 0.001).

### 3.3 Epidemiological Risk Factors Associated With Very Severe RSV/Influenza—LRTI

Univariable analysis was used to investigate the epidemiological risk factors associated with very severe LRTI associated with RSV or with influenza (Table S1). Younger age increased the odds of having very severe LRTI associated with RSV, while being exposed to smoky fuel (wood, coal, kerosene, and scavenged items) increased the likelihood of having very severe LRTI associated with influenza (Table S1).

On multivariable analysis, only age was significantly associated with very severe RSV LRTIs. For every 1 month increase in age, there was an estimated 5% reduction in the odds of having very severe RSV LRTIs (Table 2).

**TABLE 2:** Multivariable logistic regression analysis of risk factors for very severe RSV/influenza infections (very severe RSV/influenza infections are defined as RSV/influenza positive cases having SaO_2_ less than 90%).

	Very severe LRTIs associated with RSV		Very severe LRTIs associated with influenza	
	(*n* = 674)		(*n* = 149)	
Independent risk factors	aOR [95% CI]	*p*	aOR [95% CI]	*p*
Age in months	0.95 [0.91–0.97]	0.001	−0.02 [−0.10 to 0.05]	0.5
Pneumococcal carriage	0.93 [0.65–1.33]	0.7	−0.16 [−0.93 to 0.60]	0.7
Current breastfeeding	1.33 [0.82–2.14]	0.2	0.15 [−0.08 to 1.12]	0.8
Fuel type^a^	0.84 [0.58–1.22]	0.2	0.57 [−0.32 to 1.47	0.2
Cigarette smoking	0.96 [0.52–1.84]	0.9	0.73 [−0.59 to 2.07]	0.3
Number of total people in household	0.88 [0.59–1.30]	0.5	0.50 [−0.37 to 1.37]	0.3
Attending day care	1.41 [0.56–3.60]	0.5	0.17 [−2.12 to 2.46]	0.9

Abbreviations: aOR, adjusted odd ratio; 95% CI, 95% confidence interval.

^a^ Potential harmful fuel (smoky fuel): wood, coal, kerosene, scavenged items, and charcoal.

### 3.4 Risk Factor—Measles Infection

**FIGURE 2:**
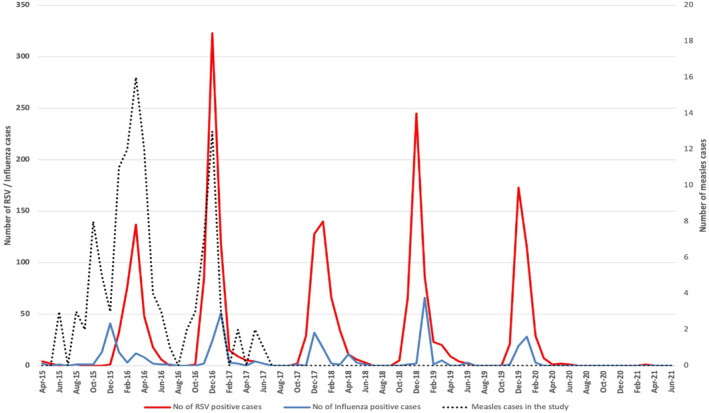
Measles cases identified among study participants.

A medical record review identified 116 study participants who had a history of measles infection during the 2015/2016 outbreak. Seventy‐five of these cases (75/116, 64.7%) had measles laboratory confirmed testing. The measles peak among these cases coincided with the relevant RSV peak (Figure 2). There was a strong positive correlation between the number of RSV cases and the number of measles cases identified among the study participants during the 2 years of measles outbreak (e.g., April 2015–September 2017), Pearson *r*(98) = 0.54, *p* < 0.0001, with measles cases explaining 30.9% of the variation in number of RSV cases during that period. The number of influenza cases was not correlated with the number of measles cases.

Among the 116 participants having a history of measles infection, 41/116 (35.3%) RSV‐positive or influenza positive cases had measles prior to admission in the current study. There were no significant differences in oxygen saturation between the group having measles infection prior to RSV or influenza infections and the group not having had prior measles infection (median 89% [IQR 89%–92%] versus median 90% [IQR 88%–91%], Mann–Whitney *p*‐value = 0.5).

### 3.5 Association Between *S.p.* Carriage and RSV/Influenza Infections

There were 2281/5705 (39.9%) cases selected for *S.p*. testing, of which 1232 had primary endpoint pneumonia and the remaining cases were randomly selected. Pneumococcal carriage was detected in 1073/2281 (47.0%) patients (Table 1); among these cases, 363 had *S.p*. and RSV codetection, 82 had *S.p*. and influenza codetection, and 15 cases had *S.p*., RSV, and influenza virus detected.

There was no significant difference in RSV detection rates between pneumococcal carriers and non‐carriers (363/1073 [33.8%] versus 400/1208 [33.1%], Fisher exact *p*‐value = 0.7). There was also no significant difference in influenza detection rates between pneumococcal carriers and non‐carriers (82/1073 [7.6%] versus 77/1280 [6.2%], Fisher exact *p*‐value = 0.2).

**FIGURE 3:**
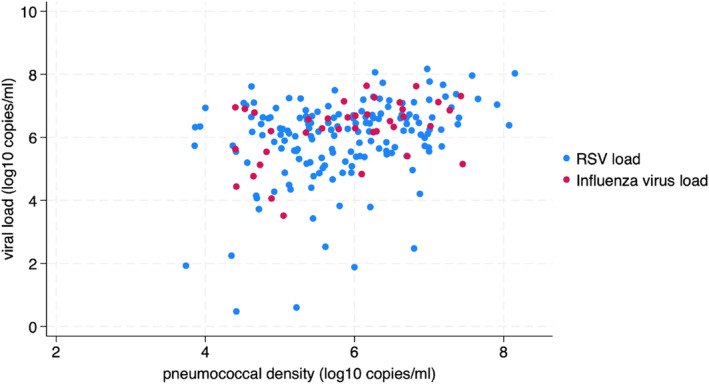
Correlations between pneumococcal density and RSV/influenza viral load. RSV load: log 10 of RSV viral load (copies/mL). Influenza virus load: log 10 of influenza viral load (copies/mL).

The density of *S.p*. carriage was positively correlated with RSV viral load (Pearson's correlation = 0.41, *p*‐value = 0.009, Figure 3) and influenza viral load (Pearson's correlation = 0.31, *p*‐value = 0.0001, Figure 3).

The proportion of children with severe LRTI among those having RSV and *S.p*. codetected was similar to the proportion of severe LRTI among cases among children with RSV alone (207/363 [57.1%] versus 213/400 [53.2%], *p*‐value = 0.3). Similarly, there was no difference in the proportions of children with very severe LRTI among children admitted with RSV alone compared to those with RSV and *S.p*. (113/400 [28.25%] versus 90/363 [24.79%], Fisher exact *p*‐value = 0.28).

Among children with influenza, there was no difference in severe LRTI rates (43/82 [52.44%] versus 40/75 [53.33%], Fisher exact *p*‐value = 1.0) as well as in very severe LRTI rates (19/82 [23.17%] versus 23/75 [30.67%], Fisher exact *p*‐value = 0.36) between children with or without pneumococcal codetection.

### 3.6 Relationship Between Disease Severity and Viral Load or *S.p.* Density

There was no evidence of associations between disease severity and viral load of RSV/influenza virus or *S.p*. density (Figures S3–S6).

## 4 Discussion

In this study, undertaken in four districts of the Mongolian capital, Ulaanbaatar, we present a comprehensive epidemiological analysis of RSV and influenza infections conducted over 6 years (April 2015–June 2021) among hospitalized LRTIs in children under 2 years of age. This analysis is set within the unique context of Mongolia.

Similar to other countries in temperate zones, distinct and stable annual RSV and influenza peaks in winter were observed throughout the six‐year study, except during the COVID‐19 pandemic, that is, December 2020–June 2021 [20]. This observation of annual peaks was also aligned to a previous report on influenza and RSV among pregnant women and children under 6 months of age in Baganuur district, which is a semi‐urban district located about 130 km away from Ulaanbaatar, in 2013–2015 [21].

The disruption of the typical seasonality of RSV and influenza during the COVID‐19 pandemic has been reported in many countries [22, 23]. This significant reduction of RSV and influenza cases was likely due to nonpharmaceutical interventions (NPIs) to limit the spread of severe acute respiratory syndrome coronavirus 2 (SARS‐CoV‐2) as well as reduced national and international travel. In Mongolia, there were lockdowns and restrictions of movements implemented in Ulaanbaatar city and the whole country at different times responding during the COVID‐19 pandemic between 2020 and 2021 [24]. Although the clinical, epidemiological, demographic data were still collected, sample collection was paused two times, December 6, 2020, to end of February 2021 and April 10 to May 8, 2021. Due to no samples collection, we could not confirm a reduction in RSV and influenza prevalence, but we observed a significant reduction of pediatric pneumonia cases.

Our data have contributed to the global effort to evaluate the burden of RSV infections [20] and RSV seasonality [25]. Of note, due to the design of the broader PCV impact study that only focused on children from 2 months of age onwards [15], our study was unable to collect epidemiological data on LRTIs associated with RSV and influenza for the vulnerable young age group of 0–1 months old. This age group is one of target populations of the newly approved RSV pediatric prevention products [26, 27]. Therefore, having data for the 0‐ to 1‐month age group would be crucial for informing future RSV prevention strategies in Mongolia. Our data also clarify RSV and influenza seasonality and describe RSV burden in Mongolia [25]. The observed predominance of RSV B in our surveillance during 2017–2018 season was aligned to the report from the INFORM study during their pilot season conducted at the same time. The INFORM study was a prospective, multicenter, global molecular epidemiology study that aimed to examine RSV subgroups distribution globally. The initial pilot season collected data in eight countries (United Kingdom, Spain, The Netherlands, Finland, Japan, Brazil, South Africa, and Australia) [20, 28]. RSV A was the predominant subgroup for the remaining study period, for example, 2015–2016, 2018–2019, and 2019–2020.

Differences in clinical severity between RSV A and RSV B have been debated [29]. In our data, a higher proportion of RSV A subgroup cases was severe. Association of severity with RSV A subgroup was mainly reported with the emergence of ON1 genotype in 2010 [30, 31]. There was also a hypothesis regarding a potential causative relationship between the substitutions in the G‐gene of RSV and disease severity, although clinical data were limited by small sample size [32]. Of note, G‐gene of RSV has been chosen as a region for genotyping in many settings, but there is a lack of consensus criteria to be used to allocate genotypes [33], challenging the interpretation of the role of genotypes in disease severity. Our future work will further investigate viral evolution and its relationship with disease severity.

In all districts, we observed a strong association between the RSV season and episodes of severe/very severe LRTIs as well as between the RSV season or influenza season and episodes of radiologically confirmed pneumonia. Our results aligned with previous studies [34] and highlight the substantial roles of the two viruses in acute lower respiratory infection burden in Mongolia.

Many studies have examined risk factors associated with severe RSV infections [19]. The most common risk factor reported across different settings was age with younger age having a higher risk of severe RSV infection [20] as our study confirmed. Our study also showed a high prevalence of potential social and epidemiological risk factors associated with severe RSV infections. However, our study could not interrogate the relationship between air pollution and severity of RSV infection. We only examined indirect factors such as informal ger housing (house type) or fuel for cooking through the study questionnaire. Air pollution is extreme during the Mongolian winter and is one of the most important country‐specific risk factors affecting health and life quality [35]. Quantified air pollution data would be needed to examine the impact of poor air quality on RSV severity.

Another important aspect of our study was the measles outbreak during 2015–2016. When our project started in April 2015, a major measles outbreak had just begun in March 2015, continuing until June 2016 [12, 13]. This large measles outbreak had two peaks in late 2015 and early 2016. The 2016 component coincided with the RSV peak of that winter. During the 2016 outbreak phase, a 10‐fold increase in measles mortality was observed, with 75% of measles‐related deaths occurring in the 0–8 months age group and 14% in the 9–11 months age group [12, 13, 36]. This underscored the vulnerability of the young age group affected during the second wave of the measles outbreak. Factors such as vitamin A deficiency and malnutrition were not found to be associated with infant measles death in a matched case–control study exploring risk factors associated with infant measles death in the second wave [13]. Although this matched case–control study did suggest nosocomial influenza B infections as a possible factor, it was not based on influenza testing of the measles hospitalized cases but rather on national influenza surveillance data and only two pathologic specimens from fatal cases [13]. Hence, our study demonstrated the positive correlation of the detection of RSV and the hospitalized measles cases, suggesting a possible interaction between measles and RSV that could contribute to higher mortality in co‐infected children [14]. The positive correlation between the number of measles cases and RSV cases could be attributed to selection bias, as only hospitalized cases for both RSV and measles were accounted for in this project. However, the susceptibility to both RSV and measles infection in the young age group is likely elevated when measles outbreaks coincide with RSV season, as observed in the Mongolia 2015–2016 measles outbreak. Infants between 3 to 9 months old, even in settings with a high measles vaccine coverage, remain vulnerable to measles infections as they have not yet received the first dose of the measles vaccine. Additionally, their maternal measles antibody titers decline below protective levels from 3 months of age [37]. This age group is highly susceptible to severe RSV infections as there are currently no RSV prevention products available in Mongolia for this specific age group.

This in turn raised a crucial question of how to protect young infants effectively, especially in the context of disrupted measles immunization programs and the impact of the COVID‐19 pandemic on RSV epidemiology in different LMICs settings [38, 39]. Measles vaccination before 6 months of age has been shown to prevent early measles infection [40, 41, 42, 43] and the measles‐associated immunosuppression, which in turn reduces susceptibility to nonmeasles pathogens including RSV [44]. In addition, measles vaccine has been shown to provide protection against other pathogens, either by cross protection, which has been demonstrated with RSV, or by nonspecific effects, which may lead to protection against other unrelated pathogens [45]. Based on our observations, we believe the impact of early measles vaccination on RSV disease should be a priority as the international community investigates earlier measles vaccine use.

The synergistic relationship between *S.p*. and RSV (or influenza) and its impact on clinical outcomes remains inconclusive. We did not observe significant differences in the RSV detection rates between children with and without *S.p*. carriage, despite a positive correlation between the density of *S.p*. carriage and the RSV (or influenza) load. Our data do not support the postulations of Weinberger et al. [46] and Ben‐Shimol et al. [47] that *S.p*. increases the severity of RSV/influenza infection or that viral infections increase the severity of *S.p*. pneumonia. This inconclusive result could be due to the limited number of samples tested for *S.p*. Testing for *S.p*. was performed only on radiologically confirmed pneumonia cases and a random sample of severe LRTIs.

Although our study population was limited to the children in four districts and only focused on severe cases, the population of the four districts covers 70% of the Ulaanbaatar population, and 69% of the total Mongolian population lives in Ulaanbaatar [48]. With very little inpatient care of children in the private sector in Mongolia, almost all sick children are managed in the participating district/referral hospitals, providing a high level of confidence that those cases should be all captured in our study for calculation of population incidence rates. The findings from our study contributed to global data on RSV season and burden [20, 25] and are valuable for informing local policy on future RSV vaccination or immunization.

## 5 Conclusion

In conclusion, our study is the first data reporting the epidemiology and RSV/influenza etiology of LRTIs hospitalizations in Mongolia, a central Asian country. Besides the potential synergistic interaction between *S.p*. and RSV, our study also highlighted the importance of preventing measles infections in the prevention of severe RSV infections. It is important to include these factors in future evaluations of RSV prevention in Mongolia as well as in any LMICs.

## Author Contributions

**Lien Anh Ha Do:** conceptualization, data curation, formal analysis, methodology, supervision, validation, writing – original draft, writing – review and editing. **Naranzul Tsedenbal:** conceptualization, data curation, formal analysis, investigation, methodology, writing – review and editing. **Chimidregzen Khishigmunkh:** conceptualization, formal analysis, investigation, methodology, writing – review and editing. **Bazarkhuu Tserendulam:** conceptualization, formal analysis, investigation, methodology, writing – review and editing. **Lkhagvadorj Altanbumba:** conceptualization, formal analysis, investigation, methodology, writing – review and editing. **Dashtseren Luvsantseren:** conceptualization, formal analysis, investigation, project administration, writing – review and editing. **Munkhchuluun Ulziibayar:** conceptualization, formal analysis, investigation, project administration, writing – review and editing. **Bujinlkham Suuri:** conceptualization, formal analysis, investigation, project administration, writing – review and editing. **Dorj Narangerel:** conceptualization, formal analysis, investigation, project administration, writing – review and editing. **Bilegtsaikhan Tsolmon:** conceptualization, formal analysis, investigation, project administration, writing – review and editing. **Sodbayar Demberelsuren:** conceptualization, formal analysis, investigation, project administration, writing – review and editing. **Casey L Pell:** conceptualization, formal analysis, investigation, project administration, writing – review and editing. **Sam Manna:** conceptualization, formal analysis, investigation, methodology, writing – review and editing. **Catherine Satzke:** conceptualization, formal analysis, investigation, methodology, writing – review and editing. **Cattram Nguyen:** conceptualization, data curation, formal analysis, methodology, writing – review and editing. **Tuya Mungun:** conceptualization, data curation, investigation, project administration, writing – review and editing. **Claire von Mollendorf:** conceptualization, data curation, formal analysis, funding acquisition, investigation, methodology, writing – review and editing. **Darmaa Badarch:** conceptualization, formal analysis, investigation, methodology, project administration, supervision, writing – review and editing. **Kim Mulholland:** conceptualization, data curation, formal analysis, funding acquisition, investigation, methodology, supervision, writing – review and editing.

## Disclosure

The funder had no role in the study design, data collection, data analysis, data interpretation, writing of the report, or the decision to submit.

## Ethics Statement

Written informed consent was obtained from parents or legal guardians of every child enrolled in the study. The study was done with the approval of the Human Research Ethics Committee (HREC) at the Royal Children's Hospital, Melbourne, HREC 33203, and at Ministry of Health Ethics Committee in Mongolia.

## Conflicts of Interest

The author declares no conflicts of interest.

### Peer Review

The peer review history for this article is available at https://www.webofscience.com/api/gateway/wos/peer‐review/10.1111/irv.13303.

## Supporting information


**Table S1** Univariate analysis of risk factors for very severe RSV/influenza infections. Very severe RSV/influenza infections are defined as RSV/influenza positive cases having SaO_2_ less than 90%.
**Figure S1.** Overview of cases of LRTIs associated with RSV or influenza during the study period. Vertical lines are the PCV introduction phased times into each district and nationally. Pre: before PCV13 introduced. Phase I: when PCV13 introduction started in SK and SB. Phase II: when PCV13 introduction started in BZ. Phase III: when PCV13 introduction started in the rest of UB including CHD (SK: Songinokhairkhan, SB: Sukhbataar, BZ: Bayanzurkh CHD: Chingeltei).
**Figure S2.** Correlations between RSV and influenza peaks and the temperature. *Temperature data were based on average monthly temperature reported by the National Agency of Meteorology and Environment Monitoring of Mongolia.
**Figure S3.** Relationship between disease severity and viral load. (A) RSV load and severe LRTIs. (B) RSV load and very severe LRTIs. (C) Influenza virus load and severe LRTIs. (D) Influenza virus load and very severe LRTIs.
**Figure S4.** Relationship between pneumonia endpoint and RSV load. PEP: primary end‐point pneumonia (radiologically confirmed pneumonia); OI: other infiltrations; no consolidations; uninterpretable for PEP.
**Figure S5.** Relationship between pneumonia endpoint and influenza load. PEP: primary end‐point pneumonia (radiologically confirmed pneumonia); OI: other infiltrations; no consolidations; uninterpretable for PEP.
**Figure S6.** Relationship between disease severity and *S. pneumoniae* density. (A) Pneumococcal density and severe LRTIs. (B) Pneumococcal density and very severe LRTIs. (C) Pneumococcal density and pneumonia endpoints. Logpneum_dens: log 10 of *S. pneumoniae* load (copies/mL); PEP: primary end‐point pneumonia (radiologically confirmed pneumonia); OI: other infiltrations; no consolidations; uninterpretable for PEP.

## Data Availability

Data are available from the corresponding author, upon reasonable request. All supporting information are freely accessible on the journal's website.
