# Supplementary material for: Respiratory Syncytial Virus and Influenza Infections in Children in Ulaanbaatar, Mongolia, 2015–2021
Source: Influenza Other Respir Viruses. 2024 May 16;18(5):e13303. doi: 10.1111/irv.13303 (PMC11099724; doi:10.1111/irv.13303)
Supplement: Supplementary file 1 — Table S1 Univariate analysis of risk factors for very severe RSV/influenza infections. Very severe RSV/influenza infections are defined as RSV/influenza positive cases having SaO2 less than 90%. Figure S1. Overview of cases of LRTIs associated with RSV or influenza during the study period. Vertical lines are the PCV introduction phased times into each district and nationally. Pre: before PCV13 introduced. Phase I: when PCV13 introduction started in SK and SB. Phase II: when PCV13 introduction started in BZ. Phase III: when PCV13 introduction started in the rest of UB including CHD (SK: Songinokhairkhan, SB: Sukhbataar, BZ: Bayanzurkh CHD: Chingeltei). Figure S2. Correlations between RSV and influenza peaks and the temperature. *Temperature data were based on average monthly temperature reported by the National Agency of Meteorology and Environment Monitoring of Mongolia. Figure S3. Relationship between disease severity and viral load. (A) RSV load and severe LRTIs. (B) RSV load and very severe LRTIs. (C) Influenza virus load and severe LRTIs. (D) Influenza virus load and very severe LRTIs. Figure S4. Relationship between pneumonia endpoint and RSV load. PEP: primary end‐point pneumonia (radiologically confirmed pneumonia); OI: other infiltrations; no consolidations; uninterpretable for PEP. Figure S5. Relationship between pneumonia endpoint and influenza load. PEP: primary end‐point pneumonia (radiologically confirmed pneumonia); OI: other infiltrations; no consolidations; uninterpretable for PEP. Figure S6. Relationship between disease severity and S. pneumoniae density. (A) Pneumococcal density and severe LRTIs. (B) Pneumococcal density and very severe LRTIs. (C) Pneumococcal density and pneumonia endpoints. Logpneum_dens: log 10 of S. pneumoniae load (copies/mL); PEP: primary end‐point pneumonia (radiologically confirmed pneumonia); OI: other infiltrations; no consolidations; uninterpretable for PEP. [file IRV-18-e13303-s001.docx]

**e.Table 1 – Univariate analysis of risk factors for very severe RSV/influenza infections (very severe RSV/influenza infections are defined as RSV/influenza positive cases having SaO2 less than 90%).**

|  | **Very severe LRTIs associated with RSV** | |  | **Very severe LRTIs associated with Influenza** | |
| --- | --- | --- | --- | --- | --- |
|  |  | |  |  | |
| Independent risk factors | **OR (95%CI)** | **p-value** | Independent risk factors | **OR (95%CI)** | **p-value** |
| Age in months (n=1,947) | ***0.94 (0.93 - 0.96)*** | ***P<0.001*** | Age in months **(**n=359) | 0.96 (0.93 - 1.01) | P=0.15 |
| Pneumococcal carriage (n=692) | 0.86 (0.61 - 1.20) | P=0.3 | Pneumococcal carriage (n=153) | 0.79 (0.38 - 1.65) | P=0.5 |
| Current breastfeeding (n=1,912) | ***1.64 (1.27 - 2.13)*** | ***P<0.001*** | Current breastfeeding (n=355) | 0.95 (0.54 - 1.65) | P=0.9 |
| Fuel type* (n=1,908) | 1.04 (0.84 - 1.29) | P=0.7 | Fuel type* (n=355) | ***3.03 (1.62 - 5.66)*** | ***P=0.001*** |
| Cigarette smoking (n=1,905) | 0.99 (0.71 - 1.37) | P=0.9 | Cigarette smoking (n=355) | 1.24 (0.55 - 2.81) | P=0.5 |
| Number of total people in household (n=1,901) | 1.07 (0.85 - 1.34) | P=0.5 | Total people in household (n=352) | 1.69 (0.92 - 3.10) | P=0.09 |
| *Being in day care (n=1,908)* | ***0.57 (0.36 - 0.96)*** | ***P=0.02*** | *Being in day care (n=355)* | 1.06 (0.44 - 2.59) | P=0.8 |

**potential harmful fuel: wood, coal, kerosene, scavenged items; charcoal.*

*OR: Odd ratio. 95% CI: 95% confidence interval*

**e.Fig 1 -** **Overview of cases of LRTIs associated with RSV or influenza during the study period.**

**
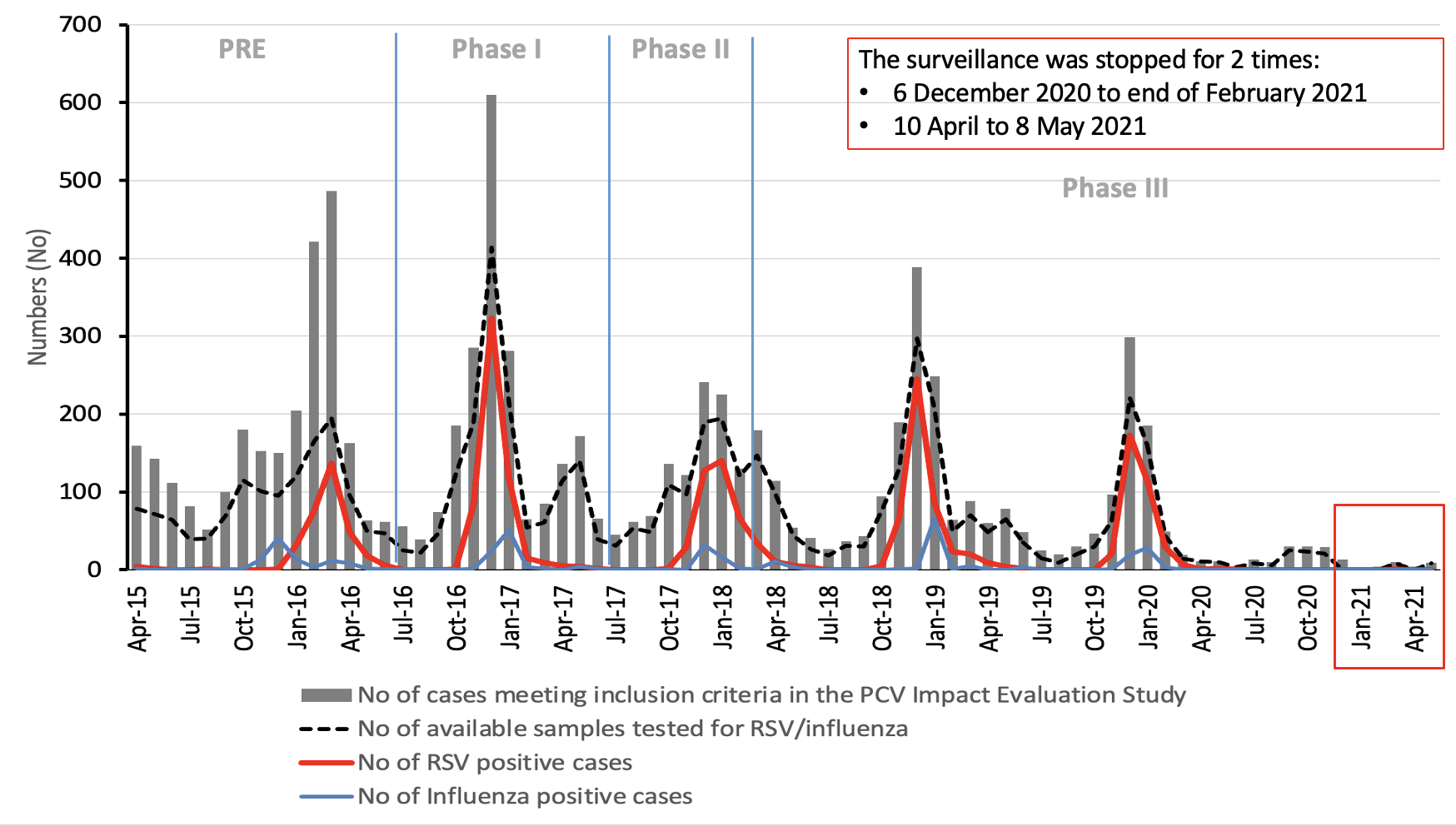
**

*Vertical lines are the PCV introduction phased times into each district and nationally.*

*Pre: before PCV13 introduced.*

*Phase I: when PCV13 introduction started in SK and SB*

*Phase II: when PCV13 introduction started in BZ*

*Phase III: when PCV13 introduction started in the rest of UB including CHD*

*(SK:Songinokhairkhan , SB: Sukhbataar, BZ:Bayanzurkh CHD:Chingeltei )*

**e.Fig 2 – Correlations between RSV and influenza peaks and the temperature.**

**
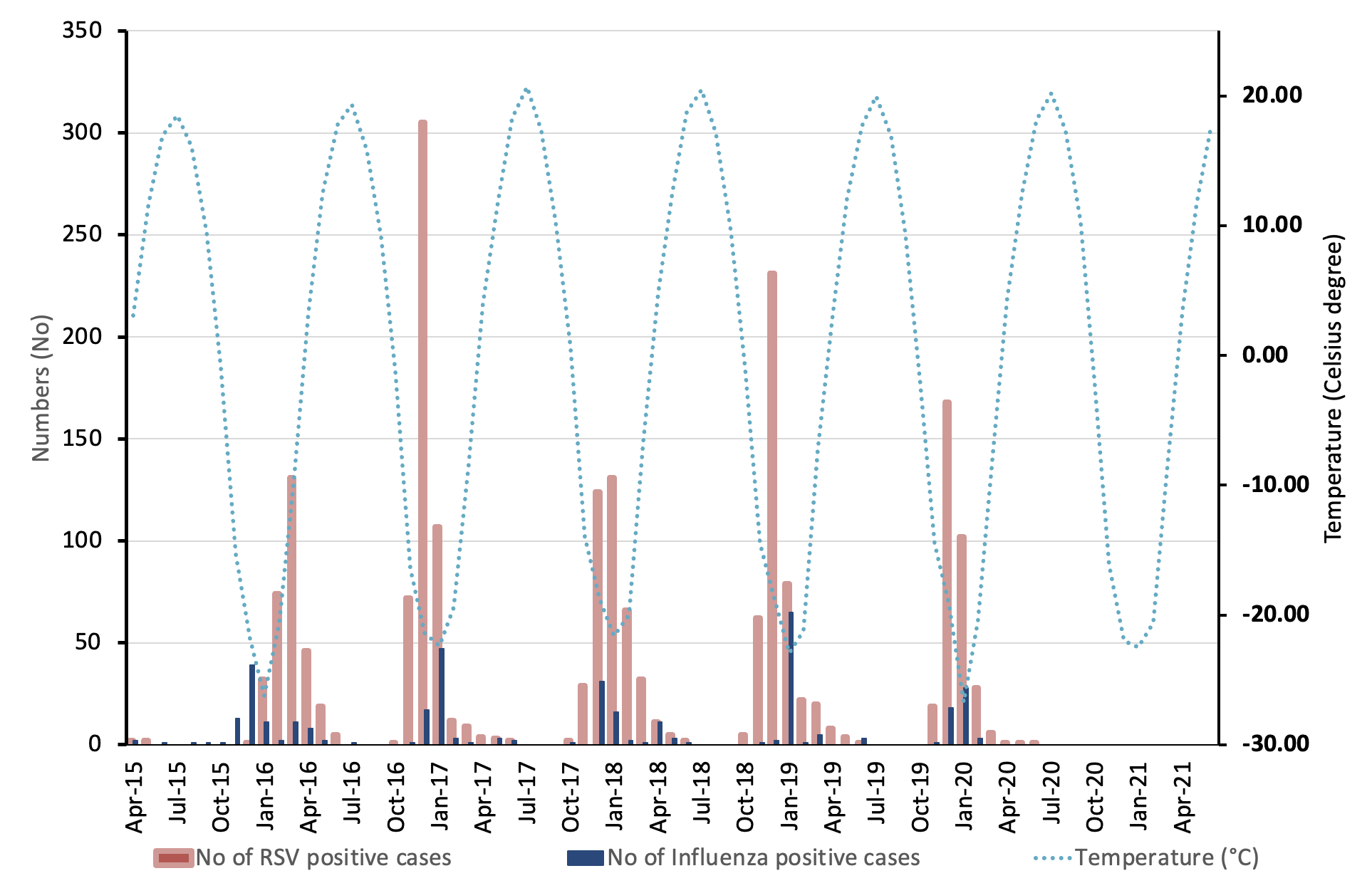
**

**Temperature data was based on average monthly temperature reported by the National Agency of Meteorology and Environment Monitoring of Mongolia*

***e.Fig 3 - Relationship between disease severity and viral load***


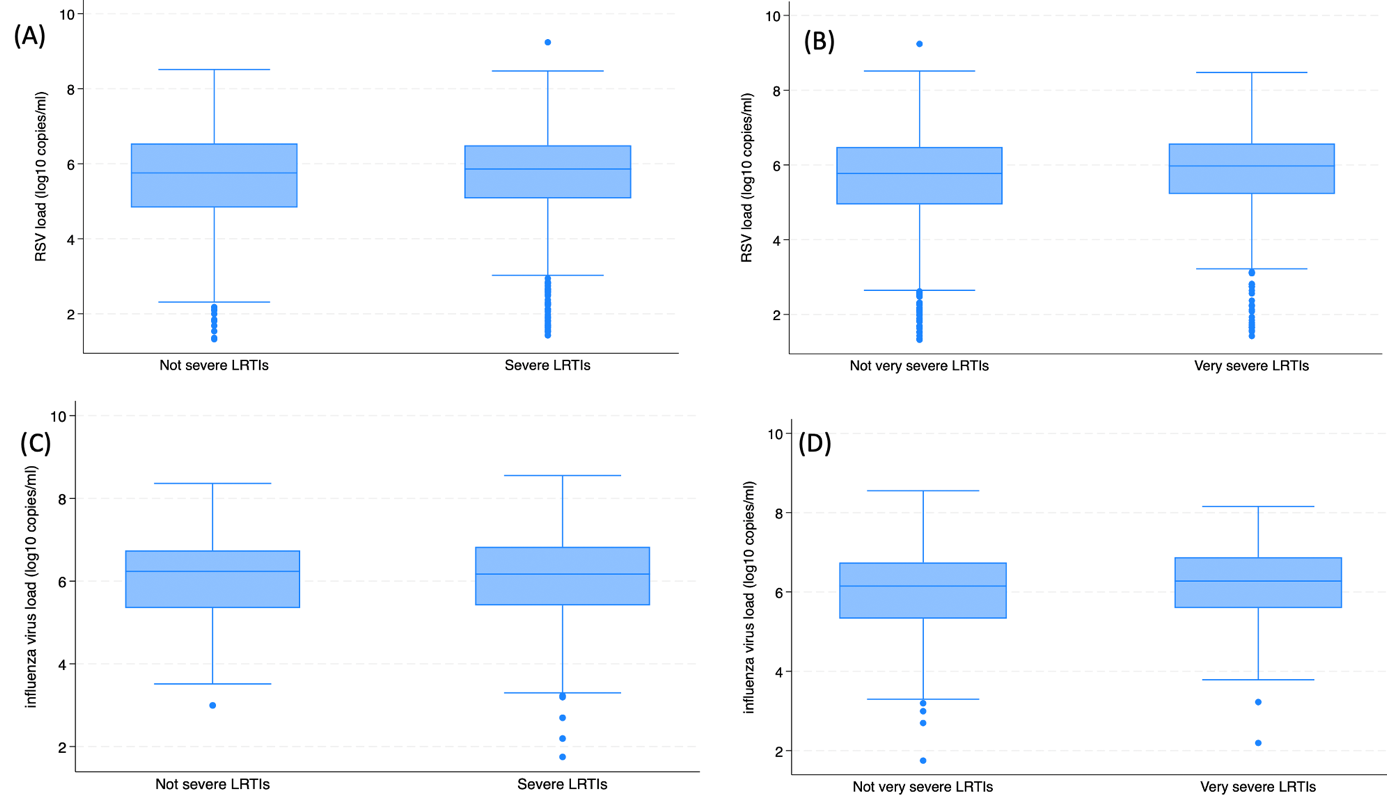


1. *RSV load and severe LRTIs*
2. *RSV load and very severe LRTIs*
3. *Influenza virus load and severe LRTIs*
4. *Influenza virus load and very severe LRTIs*

***e.Fig 4 - Relationship between pneumonia endpoint and RSV load***


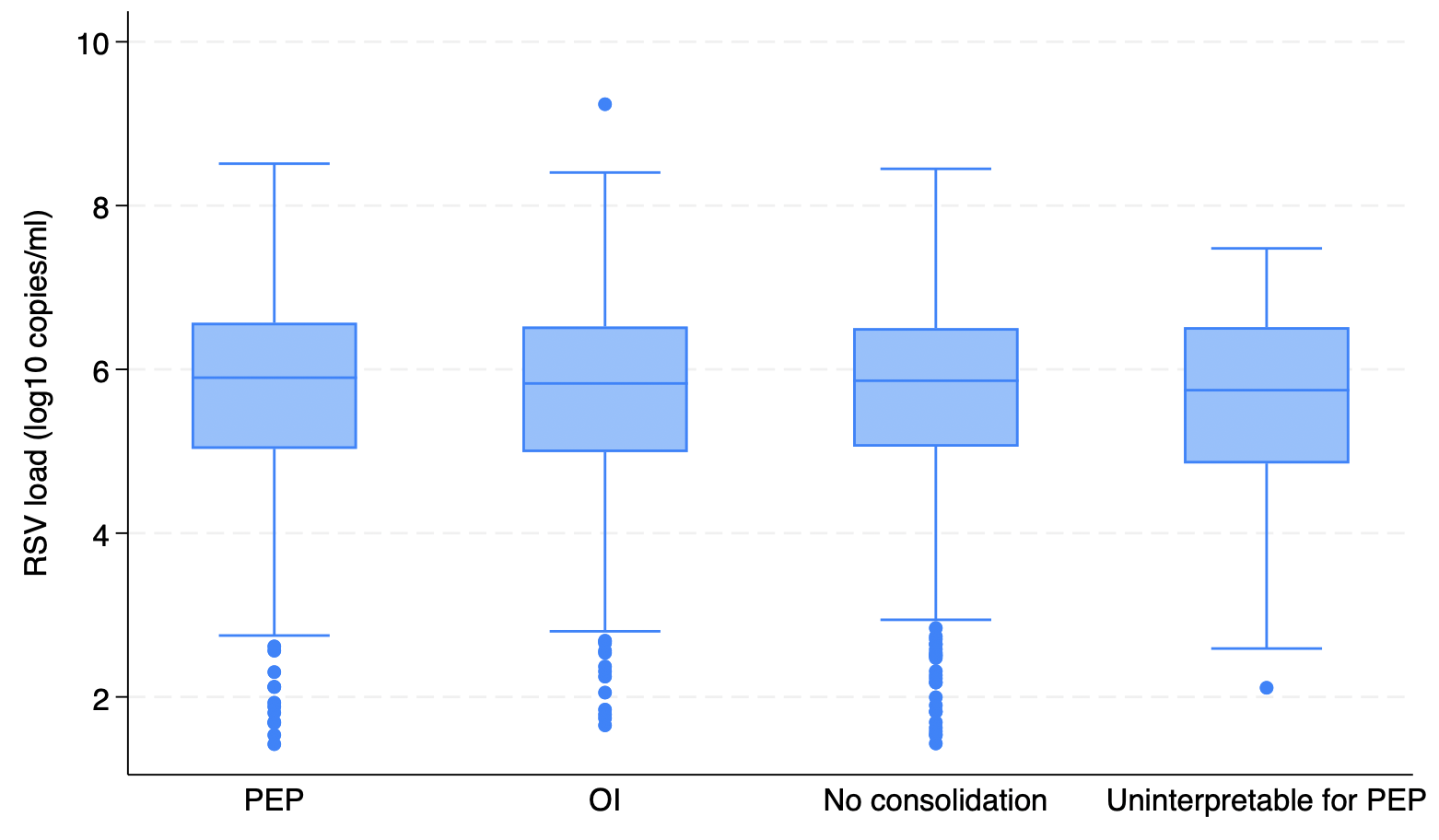


*PEP: primary end-point pneumonia (radiologically confirmed pneumonia)*

*OI: other infiltrations*

*No consolidations*

*Uninterpretable for PEP*

***e.Fig 5- Relationship between pneumonia endpoint and influenza load***

***
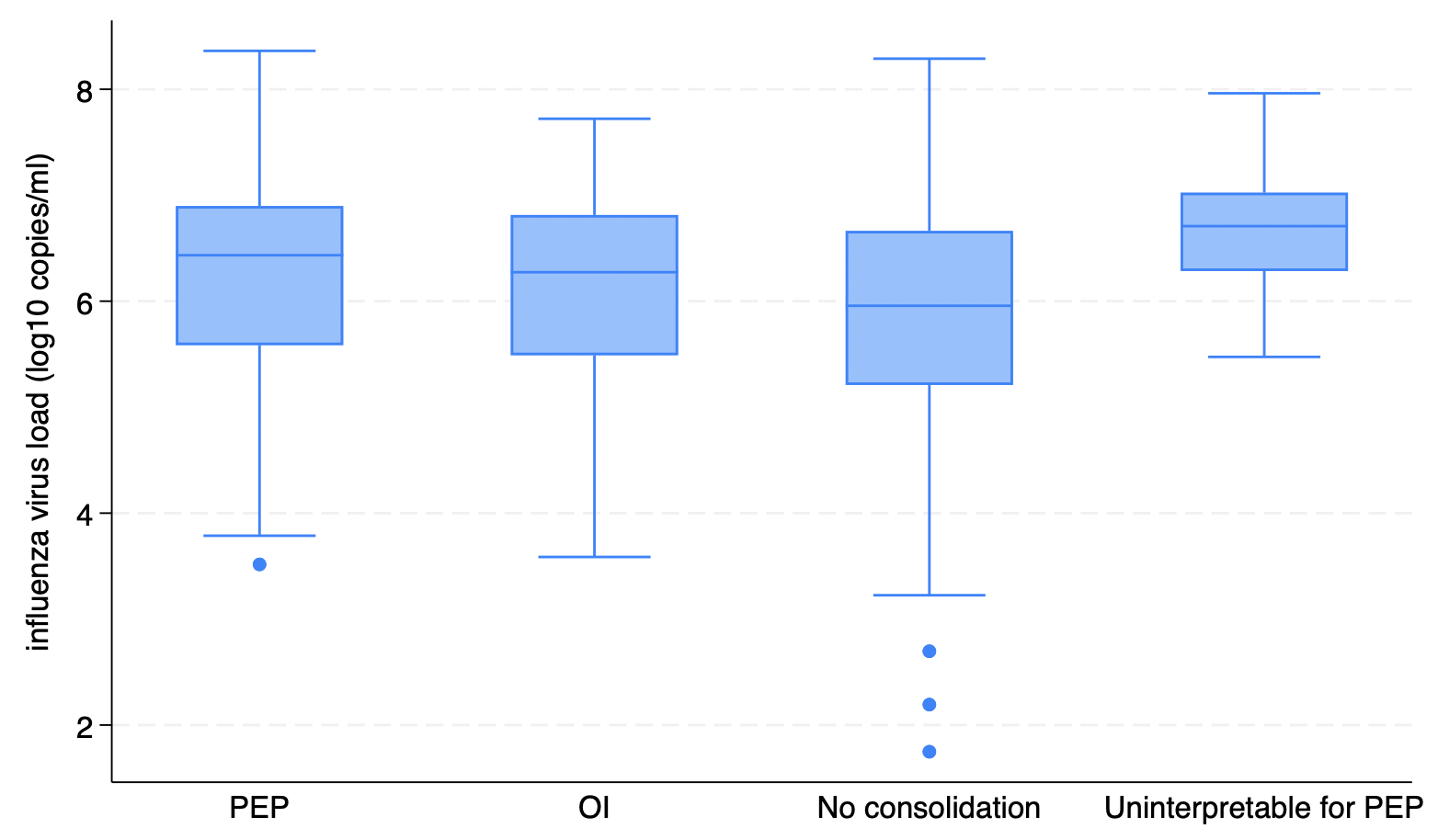
***

*PEP: primary end-point pneumonia (radiologically confirmed pneumonia)*

*OI: other infiltrations*

*No consolidations*

*Uninterpretable for PEP*

***e.Fig 6 - Relationship between disease severity and S. pneumoniae density.***

***
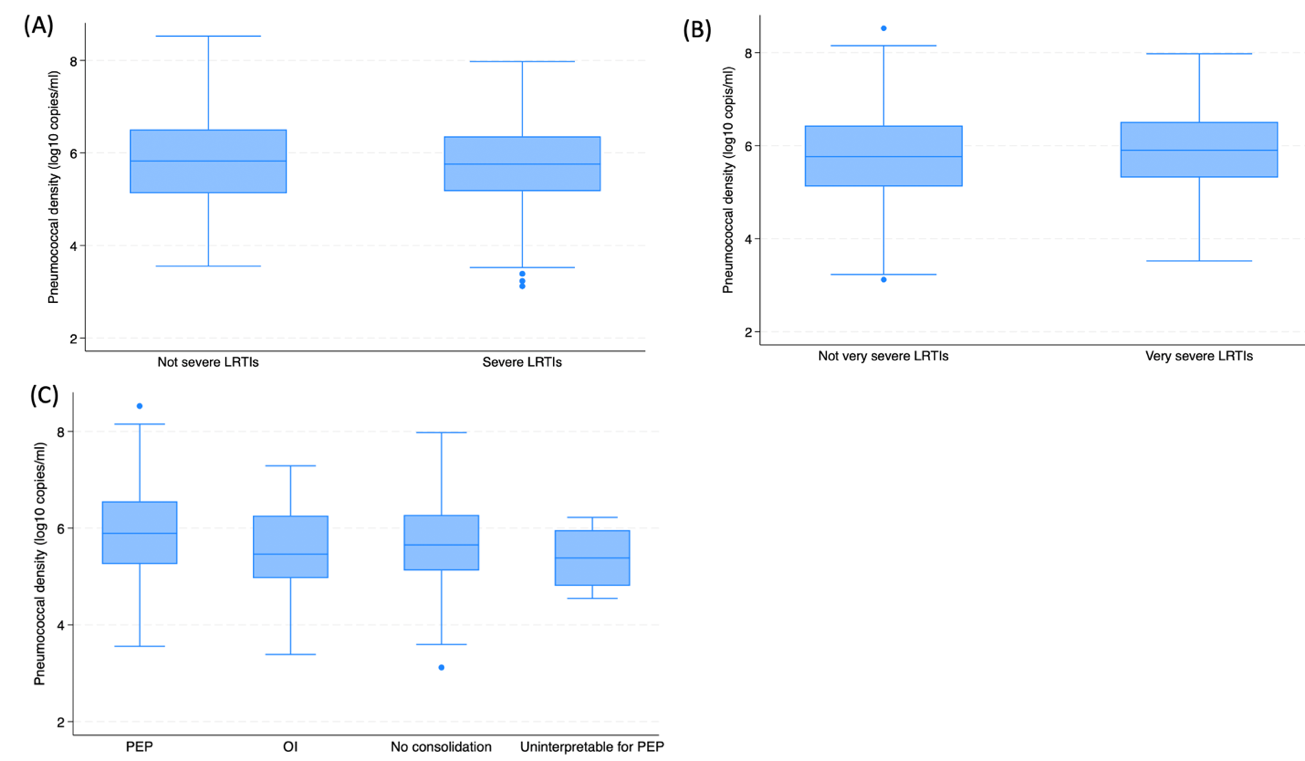
***

1. *Pneumococcal density and severe LRTIs*
2. *Pneumococcal density and very severe LRTIs*
3. *Pneumococcal density and pneumonia endpoints.*

*Logpneum_dens: log10 of S. pneumoniae load (copies/ml)*

*PEP: primary end-point pneumonia (radiologically confirmed pneumonia)*

*OI: other infiltrations*

*No consolidations*

*Uninterpretable for PEP*
